# Prolactin induces up-regulation of its cognate receptor in breast cancer cells via transcriptional activation of its generic promoter by cross-talk between ERα and STAT5

**DOI:** 10.18632/oncotarget.2376

**Published:** 2014-08-21

**Authors:** Raghuveer Kavarthapu, Chon-Hwa Tsai Morris, Maria L. Dufau

**Affiliations:** ^1^ Section on Molecular Endocrinology, Program in Developmental Endocrinology and Genetics, Eunice Kennedy Shriver National Institute for Child Health and Human Development, National Institutes of Health, Bethesda, MD

**Keywords:** prolactin, prolactin receptor, ERα, STAT5, Sp1/C/EBPβ, signal transduction, breast cancer

## Abstract

Prolactin (PRL) serves a critical role in breast cancer progression via activation of its cognate receptor. These studies reveal up-regulation of PRLR gene expression by PRL in absence of estradiol in MCF-7 and T47D breast cancer cells. PRL/PRLR via activation of STAT5 that binds a GAS-element in the PRLR gene and the participation of ERα stimulates PRLR transcription/expression. PRL/PRLR induces phosphorylation of ERα through the JAK2/PI3K/MAPK/ERK and JAK2/HER2 activated pathways. The increased recruitment of phospho-ERα, induced by PRL to Sp1 and C/EBPβ at PRLR promoter sites, is essential for PRL-induced PRLR transcription. This recruitment is prevented by blockade of PRL expression using RNA interference or ERα phosphorylation with specific inhibitors of PI3K and ERK. Direct evidence is provided for local actions of PRL, independent of estradiol, in the up-regulation of PRLR transcription/expression by an activation-loop between STAT5 and the phospho-ERα/Sp1/C/EBPβ complex with requisite participation of signaling mechanisms. PRL's central role in the up-regulation of PRLR maximizes the action of the endogenous hormone. This study offers mechanistically rational basis for invasiveness fueled by prolactin in refractory states to adjuvant therapies in breast cancer.

## INTRODUCTION

Prolactin (PRL), a polypeptide hormone of the growth hormone/cytokine family produced by lactotrophs in the anterior pituitary gland and in normal and breast tumors, exerts its diverse functions through specific transmembrane receptors. In the mammary gland prolactin induces growth and differentiation of ductal and lobuloalveolar epithelia, and is the primary hormone in the stimulation and maintenance of lactation. PRL is a potent mitogen that stimulates growth of rodent mammary tumors. In humans, PRL increases cell proliferation in mammary carcinoma cell lines, primary cultures, and breast tumor explants and it has a role in the development and progression of breast cancer [1-4]. The human prolactin receptor (hPRLR) has several forms, including the long form and short forms which are products of alternative splicing with variable lengths in their cytoplasm domains [5]. These forms are expressed in normal and breast cancer tissue, in most human breast cancer cells lines, and several other normal and in tumor tissues [5, 6]. The PRLR long form mediates all known stimulatory functions of PRL predominantly through the JAK2/STAT5 signaling pathway [7]. Independently of STAT5, PRL induces extracellular signal-regulated kinase 1/2 (ERK1/2) activity with requirement of JAK2/Src family kinases/focal adhesion kinase through phosphatidylinositol 3-kinase (PI3K)/Rac/Pak signaling pathway [8]. Moreover the participation of a Cas/cSarc/pEGFR/pSTAT5b pathway in tamoxifen resistance ERα+ T47D breast cancer cells is reversed by dominant negative Y845F EGFR and C-terminal truncated STAT5b [9].

hPRLR gene expression is controlled at the transcriptional level by multiple promoters including the generic promoter 1/exon-1 (hPIII/hEI_3_) and five human specific promoters/exons 1 [hPN1-hPN5]), which were defined and characterized in our laboratory [10]. Each promoter directs transcription/expression of a specific non-coding exon 1 (hEI_3_, hEN_1_-hEN_5_), a common non-coding exon 2, and coding exons (E3-E11). In addition to hPIII, hEI_3_ was found to contain functional elements essential for promoter activity [10]. These promoters are utilized in normal and breast cancer tissues, breast cancer cell lines, and variably in other normal and tumor tissues [11]. Our studies have shown that estrogen (E_2_) induces an increase in PRLR mRNA transcript directed by the preferentially utilized hPIII promoter which contains functional Sp1 and C/EBPβ sites but lacks an estrogen-response element. E_2_/estrogen receptorα (ERα) through complex formation with Sp1 and C/EBPβ bound at hPIII sites causes TFIIB and Pol II recruitment and increases PRLR transcription/expression over basal constitutive levels observed in the absence of E_2_ [11]. More recent studies revealed that ERα exists as constitutive homodimers in the absence of hormone and E_2_ favors its association with homodimers of Sp1 and C/EBPβ [12]. Prolactin, through its receptor, stimulates the proliferation of breast tumors and cancer cells. Increased level of PRLR receptors in breast cancer tissues suggests a role for this hormone in breast cancer [4, 13]. ERα is expressed in 75% of breast cancers in women. Inhibitors of ER and aromatase have been effectively used for therapy in breast cancer [14]. However, many breast tumors initially, or later, develop resistance to these drugs. Even after E_2_ depletion, or treatment with anti-estrogenic drugs, there is prevalence of cancer progression after the relapse period and the mechanism(s) is unclear [15]. *In vitro* and *in vivo* studies have indicated a cross-talk between prolactin and ERα in the absence of ligand [16, 17].

Thus, it is highly relevant to determine whether prolactin has a role in the up-regulation of its cognate receptor and to decipher the mechanisms involved in the regulation. PRLRs observed in tumors could maximize action(s) induced by endogenous prolactin through its receptor and be an important factor in cancer progression in the absence of E_2_. In this study, we have shown that in breast cancer cells, regulation of PRLR gene expression at the transcriptional level by its own ligand, independent of E_2_, can take place with the essential participation of the JAK2/STAT5 and mitogen-activated protein kinase (MAPK) signaling pathways. This occurs by interaction of phosphorylated ERα-generated by PRL/PRLR induced activation of signaling pathways, to transfactors associated at their hPIII promoter sites and STAT5 which binds a downstream GAS element. These findings point to a mechanism whereby PRL/PRLR could induce progression and metastasis of breast tumors that could explain persistent invasiveness in certain refractory states to adjuvant therapies.

## RESULTS

### PRL stimulation of hPRLR transcription/expression

**Figure 1:**
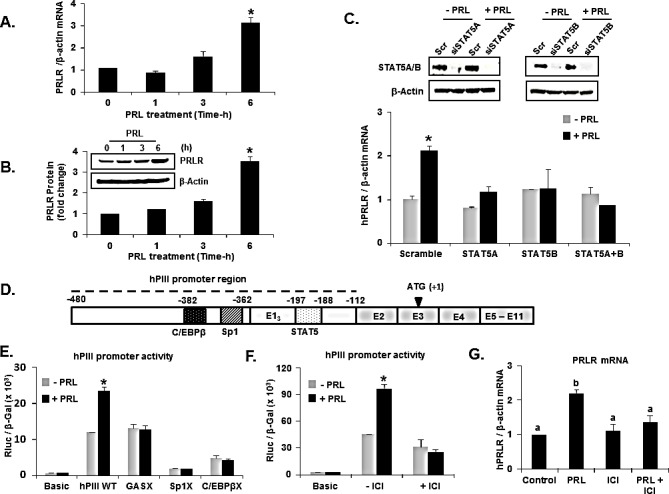
Prolactin upregulation of its cognate receptor transcription/expression Requisite participation of transcription factors. (A) Temporal expression of PRLR mRNA in response to PRL in MCF-7 cells. (B) Temporal expression of PRLR protein in response to PRL in MCF-7 cells. (C) Effect of PRL on PRLR transcripts in MCF-7 cells transfected with siRNAs: scramble (Scr), STAT5A, STATB or combination of both STAT5A and STAT5B. (D) A schematic representation of PRLR gene with the generic promoter hPIII (indicated in dotted line) including the non-coding exon-1 (hE1_3_); the common non-coding exon 2 and coding exons 3-11. (E) Effect of PRL (100 ng/ml for 6h) on PRLR promoter activity transfected with wild type hPIII/ hE1_3_ (−480/−112 bp) or constructs with Sp1 or C/EBPβ or STAT5 binding sites GAS mutated (X) or basic pGL2 vector (control) in MCF-7 cells. (F) Effect of PRL on hPIII promoter activity and mRNA in MCF-7 cells treated with an ERα antagonist, ICI 182,780 for 24 h. Results presented as relative luciferase activities (Rluc) normalized to the activities of co-transfected β-galactosidase (β-gal). (G) Effect of PRL on PRLR mRNA expression in MCF-7 cells treated with an ERα antagonist, ICI 182,780 for 24 h. Results in Figures 1 A, B, C, E, F and G are reported as the mean ± SE of three independent experiments. Asterisks indicate statistically significant increase between treated and untreated groups (*P* < 0.05). Means with a, b superscripts indicate statistically significant differences (*P* < 0.05).

In initial studies, we evaluated whether the endogenous expression of the PRLR gene governed by its generic promoter hPIII (Figure 1D) could be regulated by its cognate hormone. Real-time PCR analysis of hE1_3_ mRNA (non-coding exon 1 driven by hPIII promoter) from PRL-treated MCF-7 cells cultured in the absence of E_2_ showed a significant increase at 6 h in PRLR mRNA levels (Figure 1A) and protein expression (Figure 1B). The knock-down of STAT5A or STAT5B or both (Figure 1C) by transfection of specific siRNAs in MCF-7 cells prevented the increase in mRNA levels observed upon PRL treatment when compared those in the scrambled siRNA group (Figure 1C). This finding pointed to a regulation of the PRLR gene by PRL through STAT5A and B.

Consistent with the increase in hE1_3_ mRNA and protein, PRL treatment of cells transfected with wild type hPIII caused increase in promoter activity which was abolished by mutation of a GAS element located in non-coding exon-1 of hPIII (Figure 1E). This demonstrated the presence of a functional STAT5 site, essential for transcription of PRLR induced by PRL. Moreover, mutation of Sp1 or C/EBPβ sites at hPIII (Figure 1E) resulted in drastic reduction in promoter activity near to basic (control) value both in presence and absence of PRL. Further, the specific ERα antagonist, ICI 182,780 which promotes ER degradation, inhibited basal and PRL induced luciferase activity in MCF-7 cells, indicating involvement of ERα in PRL- induced hPIII promoter activity (Figure 1F). Moreover, ICI treatment blocked the PRL-induced transcript expression (Figure 1G). Taken together the findings demonstrated that the increase in PRLR induced by PRL was initiated at the transcriptional level with the participation of transfactors STAT5, ERα, Sp1 and C/EBPβ.

### Endogenous Recruitment of STAT5 and ERα-Sp1-C/EBPβ complex on the hPIII Promoter

**Figure 2:**
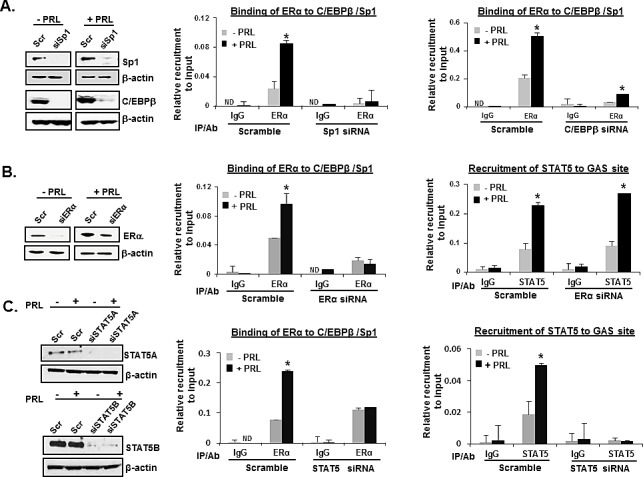
Recruitment of endogenous ERα and STAT5 onto hPIII promoter (A) ChIP assay showing the recruitment of ERα in MCF-7 cells transiently transfected with coding region of siRNAs, siSP1 (left) or C/EBPβ (right) followed by treatment with or without PRL (100 ng/ml). (B) ChIP assay showing the recruitment of ERα (left) and STAT5 (right) in MCF-7 cells transiently transfected with the coding region of SP1 siRNAs followed by treatment with or without PRL (100 ng/ml). (C) ChIP assay showing the recruitment of ERα (left) and STAT5 (right) in MCF-7 cells transiently transfected with the coding region of both STAT5A and STATB siRNAs followed by treatment with or without PRL (100 ng/ml). Scramble siRNA (Scr) was used as the positive control. The promoter region of hPIII containing SP1-C/EBPβ and STAT5 was analyzed by qPCR following immunoprecipitation (IP) with the respective antibodies. IgG was the negative control. Asterisks indicate statistically significant differences between PRL treated and untreated groups (*P* < 0.05).

**Figure 3:**
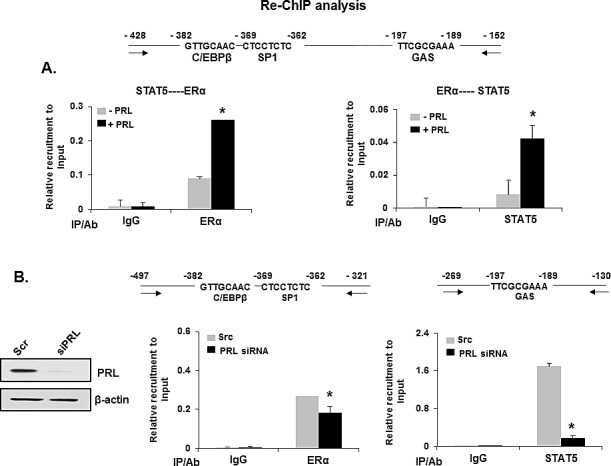
Recruitment of ERα and STAT5 and complex formation essential for transcription/expression of PRLR induced by PRL (A) Re-ChIP assay showing the recruitment and association of endogenous ERα and STAT5 onto the hPIII promoter. Re-ChIP was performed with chromatin from MCF-7 cells followed by sequential IP as indicated, with STAT5 antibody follow by ERα antibody (A, left) and in reverse sequence (A, right). (B) Effect of endogenous depletion of PRL by PRL siRNA on recruitment of endogenous ERα and STAT5 to hPIII promoter by ChIP analysis in MCF-7 cells. Effective depletion of endogenous PRL by PRL siRNA is shown in a Western blot, left versus scramble siRNA (Scr) as control, right. Asterisks indicate statistically significant difference between treated and untreated groups (*P* < 0.05).

We have previously established that E_2_ induces transcription of the PRLR gene in MCF-7 cells by direct interactions and recruitment of ERα to Sp1 and C/EBPβ bound to specific binding sites at the hPIII promoter and complex formation [11, 12]. In the present study, ChIP and RNA interference approaches were used to determine whether endogenous ERα-Sp1-C/EBPβ complex and STAT5 are recruited to their respective DNA binding sites in the PIII promoter to activate PRLR gene transcription by PRL. In MCF-7 cells, transfected with scramble siRNA, the recruitment of ERα in absence of E_2_ to Sp1 and C/EBPβ complex onto the hPIII promoter was increased by PRL treatment (Figure 2A). Basal and PRL-activated recruitment of ERα was abolished when either endogenous SP1 or C/EBPβ protein was depleted by specific siRNA (Figure 2A). Taken together, this indicated the essential role of these transfactors for the recruitment of ERα to SP1/C/EBPβ and complex formation upon PRL treatment. STAT5 recruitment to the GAS site induced by PRL treatment was not affected by ERα knockdown (Figure 2B). On the other hand PRL-induced ERα recruitment was affected when both STAT5A and STAT5B were depleted in MCF-7 cells suggesting that together both STAT5 and ERα are essential for the PRL-induced activation of hPIII promoter (Figure 2C). Re-ChIP assay performed by the sequential use of ERα and STAT5 antibodies and in reverse order revealed association of ERα and STAT5 at basal and PRL-induced promoter activation (Figure 3A). Thus, interaction of ERα with STAT5 at the promoter is required for hormonal activation of hPRLR gene transcription/expression (Figure 3A and Figures 1A-E).

It was of interest to discern whether the basal hPIII promoter activity is due to the endogenous PRL in MCF-7 cells which activates ERα in the absence of E_2_. In cells with significant reduction of endogenous prolactin by specific siRNA (Figure 3B, left) and minimal recruitment of STAT5 to the GAS element (Figure 3B, right), a significant reduction in ERα recruitment was observed (Figure 3B, left). This demonstrates the relevance of the endogenous levels of PRL in the up-regulation of PRLR observed in mammary tumors [13].

### PRL induced ERα phosphorylation through activation of PI3K/MEK/ERK pathway is required for transcriptional up-regulation of PRLR

**Figure 4:**
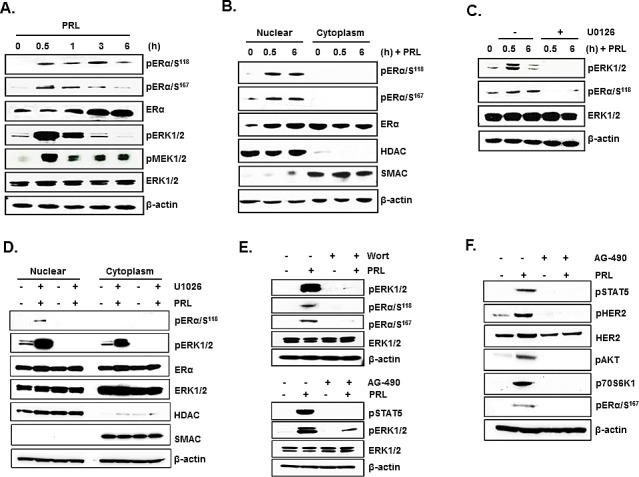
PRL induced ERα phosphorylation and nuclear translocation via the PI3K/MAPK kinase pathway (A) Western blots showing the temporal phosphorylation status of ERα (ser118 and ser167), ERK1/2 and MEK1/2 upon PRL treatment in MCF-7 cells. Total ERK1/2 and β-actin were used as loading controls. (B) Nuclear and cytoplasmic extracts isolated from MCF-7 after PRL treatment were analyzed by Western blot for p-ERα and total ERα. HDAC-1 and SMAC were monitored to validate the purity of the nuclear and cytoplasmic extracts respectively, and β-actin was used as loading control. (C) Western blots showing the effect of MEK inhibitor (U0126) on phosphorylation of ERα and ERK1/2 in MCF-7 cells treated with PRL at different time points. (D) Phosphorylation status of ERα and ERK1/2 in nuclear and cytoplasm extracts upon PRL treatment of cells for 30 min with or without addition of MEK1/2 inhibitor, U0126. (E) Western blot showing the effect of PI3 Kinase inhibitor (upper panel) and JAK inhibitor (lower panel) on phosphorylation status of ERα and ERK1/2 in MCF-7 cells treated with and without PRL. Total ERK1/2 and β-actin expression were shown as controls. (G) Effect of JAK-2 inhibitor (AG-490) on the phosphorylation of STAT5 (pSTAT5), HER2 (pHER2), AKT (pAKT), p70S6K1 (p-p70S6K1) and EK (pER) induced by PRL. The MCF-7 cells were treated either with inhibitors, U0126 (10 μM) or Wortmannin (0.5 μM) or AG-490 (50 μM) for 2h prior to the addition of PRL. Wort: Wortmannin.

**Figure 5:**
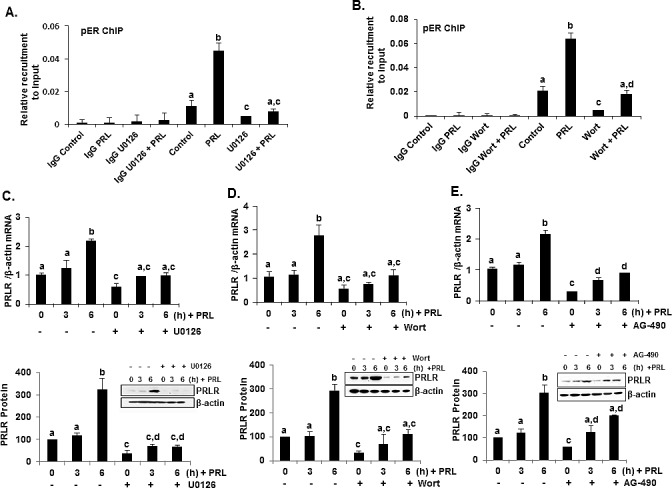
PRL induced up-regulation of hPRLR mRNA and protein expression was abolished by ERK1/2, PI3K and JAK inhibitors in MCF-7 cells (A) ChIP assay showing recruitment of pERα onto hPIII promoter in response to PRL in MCF-7 cells treated with ERK inhibitor U0126. (B) ChIP assay showing recruitment of pERα onto hPIII promoter in response to PRL in MCF-7 cells treated with PI3K inhibitor Wortmannin. Wort: Wortmannin. (C) Effect of U0126 on temporal expression of PRLR mRNA (above) and protein (below) in MCF-7 cells upon PRL treatment. (D) Effect of Wortmannin on temporal expression of PRLR mRNA (above) and protein (below) in MCF-7 cells upon PRL treatment. Wort: Wortmannin. (E) Effect of AG-490 on temporal expression of hPRLR mRNA (above) and protein (below) in MCF-7 cells upon PRL treatment. PRLR protein levels are expressed as percentage of control (control at 0h time point, 100%). Different superscripts letters indicate statistical significance differences (*P* < 0.05).

In MCF-7 cells, PRL caused rapid increase in pERα (Ser118 and 167) in total extracts after 30 min of addition to the cultures with a decline at 3h (Ser167) and at 6h (Ser118) (Figure 4A). Also, an early increase at 30 min in pERK1/2 and pMEK1/2 induced by PRL were observed in total extracts. Subsequently pERK1/2 was reduced to below control level and pMEK1/2 to slightly above control at 6h (Figure 4A). This early increase in pERα preceded the increase in ERα protein expression (Figure 4A). In nuclear extracts, PRL-induced increase in pERα (Ser118) remained elevated at 6h (Figure 4B). In contrast, no pERα was present in the cytoplasm (Figures 4B and 4D). Moreover, a major increase in pERK1/2 was observed in the nucleus and cytoplasm compartments at 30 min of PRL treatment (Figure 4D). ERα protein level in the nucleus was increased at 30 min and 6h (Figure 4B) while a simultaneous decrease was observed in the cytoplasm indicating translocation of endogenous ERα into the nucleus induced by PRL (Figure 4B). The increase in phosphorylation of ERα induced by PRL was abolished by the specific MEK inhibitor U0126 which effectively inhibited ERK1/2 phosphorylation (Figures 4C and 4D) and thus prevented the phosphorylation of ERα (Figures 4C and D). Moreover blockade of PRL-induced PI3K by the inhibitor wortmannin, prevented ERK1/2 phosphorylation required for ERα phosphorylation. This indicates that PI3K is the upstream effector of ERK1/2 activation (Figure 4E, above). PRL/PRLR induced ERK1/2 phosphorylation was abrogated by treatment with JAK2 inhibitor (AG-490) indicating JAK2 participation upstream of the PIK3/MEK/ERK pathway (Figure 4E, below). Further AG-490 treatment also abolished the phosphorylation of HER2 (tyrosine 1221/1222), AKT, p70S6K1 and ERα demonstrating PRL stimulated activation of HER2 signaling pathway through JAK2 in MCF-7 cells (Figure 4F). ChIP assays revealed PRL-induced pERα recruitment to PRLR promoter in the absence of estradiol was markedly inhibited by the treatment with both MEK1/2 and PI3K inhibitors, further demonstrating the relevance the PI3K-MAPK activation and pERα recruitment to hPIII promoter for PRL induced transcription of PRLR gene (Figures 5A and 5B). Further, the inhibition of ERK1/2, PI3K and JAK with their respective inhibitors caused marked decrease in PRL-induced PRLR mRNA and protein levels (Figures 5C, 5D and 5E).

**Figure 6:**
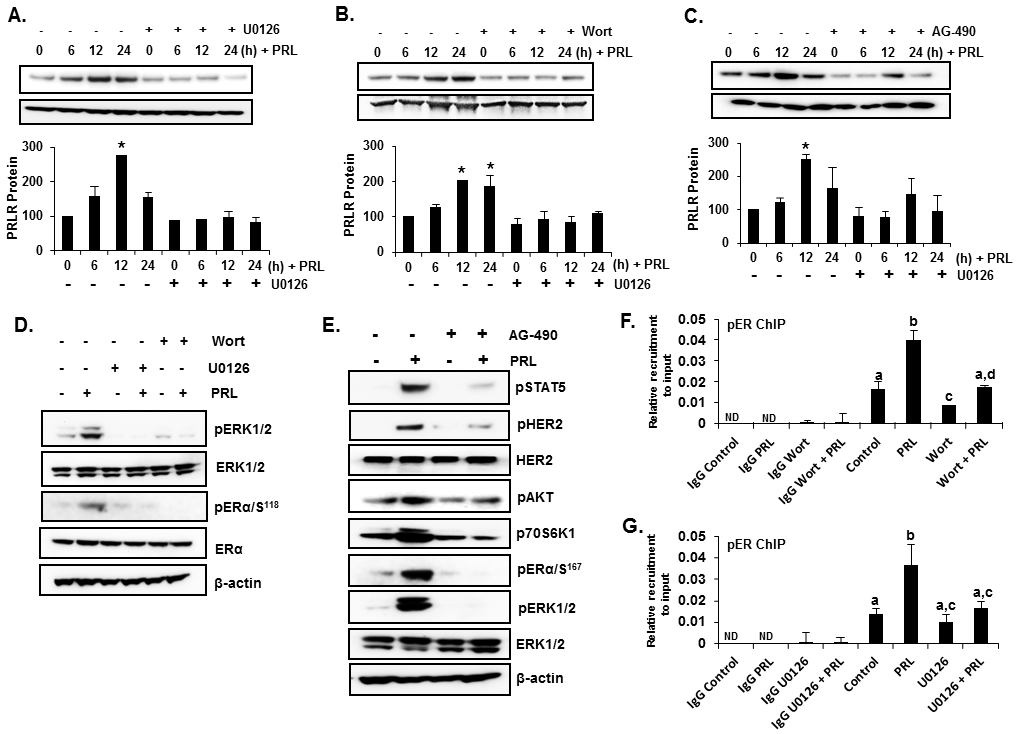
PRL induced ERα phosphorylation and up-regulation of hPRLR protein expression in T47D cells (A), (B) and (C) Lanes 1-4: -Time course of PRLR induced upregulation of PRLR. (A), (B) and (C) Lanes 4-8: -Effects of inhibitors of (A) MEK (U0126), (B) PI3K (Wortmannin), (C) JAK2 (AG-490) on temporal expression of PRLR protein in T47D cells upon PRL treatment. PRLR protein levels are expressed as percentage of control (control at 0h time point, 100%). Asterisks indicate statistically significant increase in PRLR expression when compared to 0 h time point (control) (*P* < 0.05). (D) Western blots showing the effect of MEK inhibitor (U0126) and PI3 Kinase inhibitors (Wortmannin) on phosphorylation of ERα and ERK1/2 in T47D cells treated with PRL. (E) Effect of JAK-2 inhibitor (AG-490) on the phosphorylation of STAT5 (pSTAT5), HER2 (pHER2), AKT (pAKT), p70S6K1 (p-p70S6K1) and EK (pER) induced by PRL. Total ERK1/2 and β-actin expression were shown as controls. (F) ChIP assay showing recruitment of pERα onto hPIII promoter in response to PRL in T47D cells treated with ERK inhibitor U0126. (G) ChIP assay showing recruitment of pERα onto hPIII promoter in response to PRL in T47D cells treated with PI3K inhibitor Wortmannin. The T47D cells were treated either with inhibitors, U0126 (10 μM) or Wortmannin (0.5 μM) or AG-490 (50 μM) for 2h prior to the addition of PRL. Wort: Wortmannin. Different superscripts letters indicate significanct differences (*P* < 0.05).

In T47D cells as in MCF-7 cells, PRL induced significantly PRLR protein expression levels (Figures 6A, 6B and 6C lanes 1-4). PRL induced ERα phosphorylation through activation of PI3K/MEK/ERK pathway. Upon PRL treatment a rapid increase (within 30 mins) in phosphorylation of ERK1/2 and ERα (Ser 118) was observed in T47D cells, which was abolished by both MEK1/2 and PI3K inhibitors (Figure 6D). JAK2 was also found to participate upstream of the PIK3/MEK/ERK pathway where the induction of ERK1/2 phosphorylation by PRL/PLR was abrogated by the JAK2 inhibitor (Figure 6E). The phosphorylation of HER2 (tyrosine 1221/1222), AKT, p70S6K1 and ERα (Figure 6E) was also inhibited by the JAK2 inhibitor. The increases in PRLR protein expression induced by PRL (Figures 6A, 6B and 6C lanes 1-4) were abolished by the MEK, PI3K and JAK2 inhibitors (Figures 6A, 6B and 6C lanes 5-8). These findings dissected the pathways involved in the phosphorylation of ERα whose recruitment to the PRLR promoter Sp1/C/EBPß complex is essential for the transcriptional up-regulation of the PRLR by its cognate hormone. ChIP assays further validated the PRL-induced pERα recruitment to PRLR promoter in the absence of estradiol which was markedly inhibited by the treatment with both MEK1/2 and PI3K inhibitors (Figure 6F and 6G).

## DISCUSSION

Our studies have shown that prolactin produced in breast cancer cells through intracrine activation of PRLR and via at least two signal transduction pathways, JAK2/STAT5 and JAK2/PI3K/MEK/ERK, up-regulates the human prolactin receptor. The molecular mechanism of up-regulation of this receptor by the cognate hormone has been established in this report. A functional GAS element which binds STAT5 was identified in the non-coding exon 1 of the PRLR. This exon was found in previous studies to be required for the transcriptional activity of the PIII promoter [10, 11]. Chip and ReChip studies demonstrated that DNA bound pSTAT5 associates with pERα which forms a complex to Sp1 and C/EBPß dimers bound to their respective sites at the hPIII promoter [12]. These events lead to transcriptional activation of the PRLR gene through its preferentially utilized promoter PIII and increases mRNA and receptor protein. ERα phosphorylation induced by PRL/PRLR via the PI3K/MEK/ERK pathway with upstream participation of JAK2 is required for transcriptional activation and PRLR expression. Thus, these findings provide insights into a mechanism whereby prolactin, through its PRLR and the requisite participation of the estrogen receptor, directs the up-regulation of its own receptor in the absence of estrogens.

Both STAT5A and B isoforms are expressed in MCF-7 cells and depletion of either transfactors similarly prevented increases of PRLR mRNA induced by PRL/PRLR (Figure 1C). Their requirement could be explained by their association with GAS either as tetramers A:A/B:B homodimers or A:B heterodimers. STAT5 binds specifically to γ-interferon-activated sequence (GAS) like element in the human IL-2Rα promoter as homo- or heterodimers [18]. Several studies demonstrated heterodimerization, homodimerization and tetramerization of STATs and proposed that these could provide an avenue for diversification of function by selectivity in their association with cognate elements. Moreover, association of the transcription factor dimers to their elements with tetramer formation on tandem elements enhances transcription [18]. Also, the association of STATs with a transcription factor(s) could impact gene transcription. In our studies we demonstrated interaction of STAT5 bound to a functional GAS site in non-coding exon-1 of PRLR, with pERα recruited to Sp1/C/EBPß dimers bound to their sites at the promoter. Mutations of Sp1 or C/EBPß sites prevented PRLR promoter activation via PRL/PRLR and depletion of Sp1 by siRNA abolished recruitment of ERα to the promoter. The essential role of endogenous prolactin in STAT5 and ERα recruitment to the promoter was clearly established in depletion studies using PRL siRNA. Thus, in the case of the PRLR gene the association of STAT5/ERα could serve as stabilizer of the complex which is essential for transcriptional activation of PRLR induced by PRL/PRLR in the absence of E_2_. The C-termini of STAT5A and ER were found to participate in their physical interaction [19]. Another report indicated cross-talk between STAT5B and ERα in mammary epithelial cells [20]. Although in our studies STAT5 was found to be essential for ERα recruitment to the PRLR PIII promoter, the association of STAT5 to the GAS element was independent of ERα recruitment.

Our studies demonstrated that phosphorylation of ERα induced by PRL/PRLR is a requisite for PRLR up-regulation. PRL induces rapid phosphorylation of ERα at Ser118 and Ser167 residues and nuclear translocation in MCF-7 cells in the absence of estrogen. This is consistent with a previous report which demonstrated that PRL induces ligand independent phosphorylation of ERα at Ser 118, recruitment to ERα target genes (pS2 and GREB1C) and increase of their transcription/expression in T47D cells [16]. Several kinases target Ser118 of ERα including MAPK, GSK-3, IKKα, CDK7, and mTOR/p70S6K [21]. ERα association with active MAPK in human breast tumor biopsies suggested its participation either directly or indirectly on the Ser118 phosphorylation [22]. On the other hand, Ser167 can be targeted by other kinases such as AKT, mTOR/p70S, p90RSK, and CDK2, and it can also be phosphorylated by MEK1/2, ERK1/2 [21].

In our studies we found both in MCF-7 and T47D cells significant early increases (within 30 min) of pMEK1/2 and pERK1/2 induced by PRL, coincident with those found for pERα at S118 and S167 sites. While pERα was exclusively present in the nucleus, pERK1/2 was distributed in nucleus and cytoplasm. ERα phosphorylation induced by prolactin was abolished by preincubation of cells with MEK inhibitor (U0126) or PI3K inhibitor (Wortmannin) indicating the involvement of PI3K/MAPK signaling pathway in the PRL /PRLR induced up-regulation of PRLR. Moreover, PRL-induced recruitment of pERα to the promoter, required for its association to STAT5 and the Sp1/CEBPß complex, was abolished by the MEK1/2 and PI3K inhibitors, consequently the increase expression of PRLR was not observed. PRL through PRLR can simultaneously activate the JAK/STAT5, PI3K-AKT, and PI3K-MAPK signaling pathways. Further, in our studies incubation of cells with the specific JAK2 inhibitor AG-490 completely abolished STAT5 phosphorylation and decreased ERK1/2 phosphorylation by 80%. The minor residual ERK1/2 phosphorylation could be contributed by PRL/growth factor associated pathways. Our findings indicated an upstream requirement of JAK2, which accounted for the most activation of the pathway leading ERK1/2 phosphorylation induced by PRL.

Taken together, our studies have demonstrated an increase in expression of PRLR receptors in breast cancer cells induced by endogenous PRL/PRLR action via STAT5 interaction with pERα and complex formation with Sp1/C/EBPβ at the PRLR promoter in the absence of estrogen (Figure 5). The participation of the JAK2/PI3K/MEK/ERK pathway is required for phosphorylation of ERα a requisite for its recruitment to the PRLR promoter. These studies are centered on the mechanism of PRL action and ERα activation in the absence of E_2_. The expression of its own receptor could be a factor in resistance to adjuvant therapy with aromatase inhibitor in ERα^+^/human epidermal growth factor receptor 2 (HER2)^−^ breast cancer. It also could impact ERα^+^/HER2^+^ tumors, which account for about 10% of the ER^+^ breast cancers, since HER2 can be phosphorylated at tyrosine residues via JAK2 by autocrine secretion of PRL in breast tumors [23]. An accepted mechanism of resistance to endocrine therapy is overexpression and activation of the HER2 proto-oncogene [24]. HER2 has no known ligand and is constitutively active for heterodimerization with other members of HER receptor family. The impact of PRL/PRLR/JAK2 on HER2 active conformation (constitutive) cannot be excluded. Heterodimers of HER2/HER3 constitutively or ligand stimulated cause activation of PI3K/AKT/mTOR pathway and phosphorylates P70S6 kinase 1 which in turn phosphorylates the activation domain of ERα [25]. In addition the MEK/ERK1/2 is also activated by HER2 heterodimers [24]. Moreover, we showed in this study both in MCF-7 and T47D cells which express HER2 that PRL/PRLR induces HER2 phosphorylation via JAK2, activating the downstream PI3K/AKT pathway. This crosstalk could also enhance phosphorylation of ERα, its recruitment to the PRLR promoter and PRLR transcription/expression. Disregulation of the PI3K pathway has been linked with resistance to adjuvant hormone and HER2 target therapies. Treatments at various levels of the PI3K pathway with Pan PI3K or mTOR inhibitors were effective in restoration of hormone sensitivity and transtuzumab resistance [24]. Moreover, *in vitro* studies using ERα^+^ letrozole resistant MCF-7 cells demonstrated that resistance was more effectively abrogated by targeting PI3K/AKT/mTOR and MEK/ERK pathways with inhibitors of both pathways [14, 26, 27]. These treatments could effectively inhibit PRL/PRLR induced increase of its cognate receptor.

Incorporation of therapeutic agents that inhibit the function of the hormone PRL or the PRLR receptor with those targeting the various signal transduction pathways, including those resulting from activation of HER2 could improve effectiveness in reversing resistance in breast cancer. Given the effective inhibition of PRLR up-regulation by ICI observed in our studies, a combination therapy targeting directly ER and PRLR could offer an additional approach to eliminate constitutive activation of ER and of PRLR by endogenous PRL and circumvent resistance. These studies providing mechanistic insights into the up-regulation of the PRLR by its endogenous cognate hormone and receptor indicate the relevance of their participation in resistance to adjuvant therapies and further the basis for the treatment of refractory states in ERα^+^ breast cancers.

**Figure 7:**
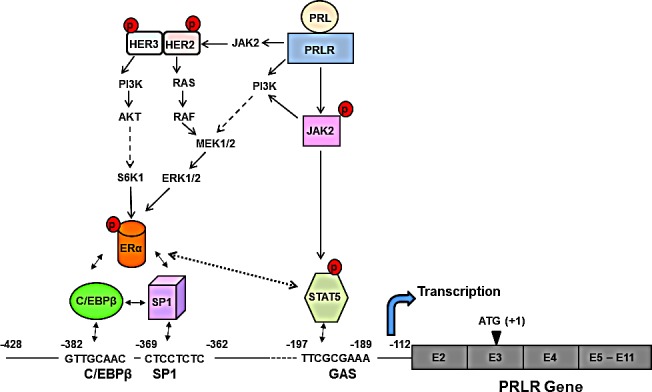
Proposed mechanism of the upregulation of hPRLR induced by its cognate hormone PRL produced locally in the normal and tumoral breast activates the JAK2/STAT5 pathway via the long form of the PRLR. The pSTAT5 bound to a GAS element located in the non-coding exon 1 of the PRLR associates with pERα recruited to Sp1 and C/EBPβ bound to their sites at the hPRLR promoter causing increase hPRLR transcription, mRNA and protein. PRL/PRLR, independent of estrogen, induces ERα phosphorylation (via the JAK2/PI3K-MEK-ERK pathway), nuclear translocation, and complex formation by its recruitment to Sp1/C/EBPβ bound to their DNA sites at the promoter and transcriptional activation. PRL/PRLR activates JAK2 which phosphorylates HER2 and activates several pathways which in turn may cause phosphorylation of ERα. The series of molecular events induced by endogenous PRL via PRLR causing up-regulation of its cognate receptor could participate in breast cancer progression and explain resistance to endocrine therapy.

## METHODS

### Reagents and Antibodies

Phenol free PRMI 1640 medium and charcoal treated fetal bovine serum were obtained from Atlanta Biologicals. The antibodies against ERα, STAT5A, STAT5B, STAT5, Sp1, HDAC-1, SMAC, β-actin and inhibitors of PI3K, Wortmannin, and JAK2, AG-490, were obtained from Santa Cruz Biotechnology (Santa Cruz, CA). Phospho (p)-ERα (Ser118 and Ser167), pSTAT5, pERK1/2, pMEK1/2, pHER2 (Tyr 1221/1222), HER2 and ERK1/2 antibodies and U1026 (MEK1/2 inhibitor) were purchased from Cell Signaling. ICI 182,780 (Fulvestrant) and β-actin antibody were obtained from Sigma-Aldrich. Human PRL antibody and human PRL were obtained from National Hormone and Peptide Program, Harbor-UCLA Med. Ctr., Torrance, CA 90502.

### Cell culture, transfection, and reporter gene assay

MCF-7A2 (MCF-7) (ER^+^ and HER2^+^) (gift from E. Berleth, C. Roswell Park Cancer Institute, New York, NY) and T47D (ER+ and HER2+) breast cancer cells obtained from ATCC were maintained in RPMI 1640 medium supplemented with 10% charcoal-treated fetal bovine serum. Cells were cultured in six-well plates under steroid-free conditions in PRPMI phenol red-free media without supplemental growth factors with 5% and 1% charcoal-treated fetal bovine serum for 2 days at each serum concentration. The PRLR hPIII promoter/non-coding exon 1 (hE1_3_) reporter pGL2 gene construct (bp −480/−112) containing C/EBPβ, Sp1 sites in hPIII and putative STAT5 binding site in hE1_3_, and other constructs with the sites mutated, and pGL2 empty vector were used for transient transfection in cells using Lipofectamine 2000 reagent (Invitrogen) as described previously [11]. After 18 h, cells were treated with PRL (100 ng/ml) for 6 h and harvested for the determination of luciferase activity (Promega).

### Real Time RT-PCR

Total RNA was isolated from MCF-7 cells treated with or without PRL (100 ng/ml) at various times. Total RNA (1 μg) was used for reverse transcription reaction using Superscript III First Strand RT synthesis kit (Invitrogen). One-step real-time quantitative reverse transcription-PCR was carried out using SYBR Green QPCR Master Mix and an ABI 7500 sequence detection system (Applied Biosynthesis, Foster City, CA). Specific primers for amplification of hPRLR mRNA transcribed from promoter hPIII were a forward primer derived from non-coding exon 1 (hE1_3_) (−204/−180) and reverse primer from sequences located in common hE3 (+2/+22). Samples were normalized to the level of β-actin mRNA.

### Preparation of Whole Cell Lysate and Nuclear Protein and Western Blot Analysis

Whole cell lysates from MCF-7 and T47D cells were extracted using RIPA lysis buffer (Thermo Scientific, Rockford, IL). The nuclear and cytosolic proteins from MCF-7 cells cultured in presence and absence of PRL (100 ng/ml) or treated with ICI were isolated using NE-PER Nuclear and Cytoplasmic Extraction kit (Thermo Scientific) in presence of 1x protease and phosphatase inhibitor cocktail (Thermo Scientific). Samples were resolved on NuPAGE 4 to 12% Bis-Tris gradient gels and transferred to nitrocellulose membranes (Invitrogen). Membranes were blocked with 5% skimmed milk powder in phosphate buffer saline and incubated with primary antibodies. Immunosignals were detected by super-signal chemiluminescence system (Pierce).

### siRNA Analysis

Two sets of validated siRNAs designed to knock-down the endogenous expression of STAT5A, STAT5B, Sp1, ERα, PRL and scrambled control siRNA were purchased from Ambion (Life technologies). The MCF-7 cells were transfected with 25 nM siRNA using siPORTNeoFX reagent (Life technologies) as described previously [12]. After 24 h of siRNA transfection in 10% CTS, cells were grown in 5% CTS for 2 days and 1% CTS for another 2 days. The cells were harvested for Total RNA extraction or Chip assays after treatment with PRL (100 ng/ml) in serum free medium for 6 h. All siRNA sequences are available in Table 1.

**Table 1:** List of validated siRNAs used for gene knockdown and their sequence information

Gene Name	siRNA Sequence
Sp1	GCAACAUGGGAAUUAUGAATT GGCAGACCUUUACAACUCAAG
C/EBPβ	GGCCCU GAGUAAUCGCUUTT CCGCCUGCCUUUAAAUCCATT
ERα	CAGGCACAUGAGUAACAAATT CGAGUAUGAUCCUACCAGATT
STAT5A	AUGGAUAUGUGAAACCACATT GCGCUUUAGUGACUCAGAAAT
STAT5B	CACCCGCAAUGAUUACAGUTT GCATCACCATTGCTTGGAAGT
PRL	GCGAAUUCGAUAAACGGUATT GCATATTGCGATCCTGGAATG
Scramble	AATTCTCCGAACGTGTCACGT

### Chromatin Immunoprecipitation (ChIP) Assay

ChIP assays were performed using MAGnify^TM^ Chromatin Immunoprecipitation system from Invitrogen following the manufacturer's protocol as previously described [12]. The relative binding of proteins of interest to the hPIII promoter was quantitatively analyzed by real-time PCR assay of the precipitated DNA and input DNA using SYBER Green Master Mix in an ABI 7500 sequence detection system. The primers utilized for amplification of the hPRLR gene promoter sequence that spans the GAS site were located at −269/−248 (forward) and −154/−130 (reverse). Primers for both Sp1 and C/EBPß were located at −497/−471 (forward) and −321/296 (reverse).

### Re-ChIP Assay

Immunocomplexes from the primary immunoprecipitation were re-immunoprecipitated with different specific antibodies. Briefly 5 × 10^6^ MCF-7 cells were used to prepare the soluble chromatin by sonication and immunoprecipitation was carried out at 4°C overnight with specific antibodies to ERα or STAT5 or IgG. Following immunoprecipitation, protein A-Sepharose beads were added and incubated for 2 h at 4°C. After several rounds of washing the immunocomplex was added to the Dynabeads pre-coupled with ERα or STAT5 antibodies and incubated overnight at 4°C for second round of immunoprecipitation. After washing steps, the antibody-Dynabeads complexes were reverse-crosslinked and DNA was purified with DNA purification magnetic beads. The relative binding of proteins of interest to the hPIII promoter was quantitatively analyzed by real-time PCR as mentioned above. The primers utilized for qPCR were located at −429/−403 (forward) and −173/−151 (reverse).

### Statistical analysis

Data are presented as the mean ± SE of three independent experiments performed in triplicate. The significance of the differences between groups in the presence and absence of PRL was determined by the multiple Tukey's multiple-comparison test (one-way ANOVA analysis of variance) using the Prism software program (GraphPad Software, Inc, San Diego, California). Statistical significance was considered as *P* < 0.05.

## Abbreviations

PRL: prolactin
PRLR: prolactin receptor
JAK2: janus kinase 2
STAT5: signal transducer and activator of transcription 5
ERα: estrogen receptor α
SP1: specificity protein 1
C/EBPβ: CCAAT-enhancer-binding protein
PI3K: Phosphatidylinositide 3-kinase
ERK1/2: extracellular signal-regulated kinase 1/2
MEK1/2: mitogen-activated protein kinase
HER2: human epidermal growth factor receptor 2
RAS: Rat sarcoma
C-RAF: rapidly accelerated fibrosarcoma (protein kinase)
AKT: protein kinase B
mTOR: mammalian target of rapamycin
S6K1: Ribosomal protein S6 kinase-1
